# Understanding menstrual cycle effects on suicide will require prospective studies of suicidal thoughts and behaviors in premenstrual disorders

**DOI:** 10.1186/s12916-021-02010-8

**Published:** 2021-06-03

**Authors:** Sarah A. Owens, Tory A. Eisenlohr-Moul

**Affiliations:** 1 Department of Psychology and Neuroscience, University of North Carolina at Chapel Hill, 235 E. Cameron Avenue, Chapel Hill, NC 27599-3270 USA; 2 Departments of Psychiatry and Psychology, University of Illinois at Chicago, Neuropsychiatric Institute, 912 S Wood St, South Tower, MC 913, Chicago, IL 60612 USA

## Background

The past 50 years of suicide research have produced a small set of distal, between-person factors that predict suicide attempts only slightly better than chance, and rates of suicide death have not declined over the same timeframe [1]. In order to prevent suicide, it is critical to identify time-varying factors that predict acute changes in risk, allowing for targeted intervention in the days and weeks where individuals are most vulnerable. Ample cross-sectional data indicate that some individuals experience menstrual cycle-related changes in suicidal behavior and associated risk factors [2], suggesting that hormone fluctuations across the menstrual cycle may be one predictable time-varying trigger for acute increases in suicide risk. In a field that has largely fallen short of identifying such triggers, the present study makes an important contribution by highlighting a vulnerable group who may experience a reliable pattern of change in risk. However, this area of research faces several barriers to advancement, including the need for replication in prospectively confirmed premenstrual dysphoric disorder (PMDD) and the urgent need to integrate suicidality into our scientific and clinical conceptualizations of PMDD.

## Advancing the study of menstrual cycle changes in suicide risk: future directions

Yang et al. add to the field of suicide research by examining whether provisional clinical PMDD diagnoses are associated with greater suicide risk among a large, population-based cohort followed for over a decade [3]. This study underscores the importance of identifying *who* is at risk for menstrual cycle-related changes in suicidal behavior, given that there are marked between-person differences in the extent to which ovarian hormone fluctuations elicit changes in affect and behavior [4]. Although it is unlikely that most diagnoses in this study were confirmed with daily ratings [5], these data are consistent with cross-sectional investigations finding that retrospectively-reported PMDD is linked with suicide risk.

Unfortunately, PMDD diagnoses based on retrospective self-report or interview at a single time point have inadequate specificity, as the majority of cases identified in this manner fail to demonstrate significant cyclical symptoms when tracked daily across the cycle [6]. Sensitivity may also be a concern, as underreporting of cyclicity has been reported in psychiatric samples [7]. The DSM-5 acknowledges these measurement problems by requiring two cycles of daily ratings for diagnosis; nevertheless, due to the time-intensive nature of prospective assessment, nearly 90% of clinicians who treat premenstrual disorders rely on a single time point interview for diagnosis [5]. Notably, this method is also inadequate to distinguish between PMDD and premenstrual exacerbation of an underlying disorder (i.e., without follicular symptom remission), which is likely more common than PMDD and may not respond to evidence-based PMDD treatments [8]. Given the limited validity of retrospective diagnoses, the association between provisional PMDD diagnosis and suicide risk in the present study may be an over- or under-estimation of true effects.

While the measurement problem is clear, an accessible solution has yet to be developed. Algorithms for diagnosing PMDD based on daily ratings in research are available (C-PASS; [6]), but these are not yet user-friendly enough for routine clinical use. Methodological innovation is therefore needed to streamline rigorous PMDD diagnostic evaluations. For example, while many smartphone apps allow for daily symptom tracking, most track symptom presence rather than severity, and none currently include all DSM-5 criteria for PMDD or integrate clearly defined scoring algorithms. Creation of automated systems for collection of daily ratings as well as standardized scoring and reporting of results to clinicians [6] should be a priority.

In addition, the present results underscore the need for experiments to determine the pathophysiological mechanisms underlying menstrual cycle changes in suicide risk among hormone-sensitive individuals. In this study, the association between provisional PMDD diagnosis and suicide risk remained in spite of the fact that many individuals were receiving PMDD treatment (e.g., SSRIs). This may be due to treatment non-response, as only about 60% of individuals with prospectively diagnosed PMDD respond to SSRIs, the first line approach [9]. In order to develop more effective treatments, we need a more granular understanding of both the physiological mechanisms by which hormone flux triggers changes in affect and increased risk, and the cognitive and psychosocial mechanisms that lead to corresponding increases in suicidal behavior. Use of intensive longitudinal designs as well as experimental studies will be critical to uncover and target these mechanisms.

Given that the status quo in the field of PMDD is to exclude participants with suicidal ideation and behavior, perhaps the most important implication of these findings is that it is critical that future studies of PMDD include these participants and incorporate daily measures of suicidal thoughts and behaviors. While many researchers may be discouraged from including these patients and studying these constructs due to concerns about liability, guidelines and algorithms are available that can streamline and standardize ethical protocols for suicide risk assessment and management [10]. Ideally, both lifetime history and daily measures of suicidal thoughts and behaviors should be included in longitudinal PMDD studies.

## Conclusions

Should the link between PMDD and suicide risk replicate in a series of longitudinal studies with prospective daily ratings, the DSM working group for PMDD should consider the inclusion of suicidal ideation and behavior in the criteria list for the disorder. If cyclical suicidality is indeed a common feature of PMDD, then inclusion of this symptom in subsequent criteria sets could (1) reduce misdiagnosis (e.g., if clinicians mistakenly believe that any suicidality observed in PMDD must necessarily indicate an alternative psychiatric disorder) and (2) save lives by increasing clinician awareness, assessment, and intervention around recurrent cyclical suicidality. However, before these possibilities can be considered, there must be an increased willingness on the part of scientists to study suicidal thoughts and behaviors as a “real time” daily outcome of PMDD, so that sufficient prospective evidence can accumulate on this topic prior to the next revision of the DSM PMDD diagnosis.

## Data Availability

Not applicable.

## References

[1] Franklin JC, Ribeiro JD, Fox KR, Bentley KH, Kleiman EM, Huang X, Musacchio KM, Jaroszewski AC, Chang BP, Nock MK (2017). Risk factors for suicidal thoughts and behaviors: a meta-analysis of 50 years of research. Psychol Bull.

[2] Owens SA, Eisenlohr-Moul TA (2018). Suicide risk and the menstrual cycle: a review of candidate RDoC mechanisms. Curr Psychiatry Rep.

[3] Yang Q, et al. Clinical indications of premenstrual disorders and subsequent risk of injury: a population-based cohort study in Sweden. BMC Med. 2021. 10.1186/s12916-021-01989-4. 10.1186/s12916-021-01989-4 PMC8152351 34034729

[4] Wei S-M, Schiller CE, Schmidt PJ, Rubinow DR (2018). The role of ovarian steroids in affective disorders. Curr Opin Behav Sci.

[5] Craner JR, Sigmon ST, McGillicuddy ML (2014). Does a disconnect occur between research and practice for Premenstrual Dysphoric Disorder (PMDD) diagnostic procedures?. Women Health.

[6] Eisenlohr-Moul TA, Girdler SS, Schmalenberger KM, Dawson DN, Surana P, Johnson JL, Rubinow DR (2016). Toward the reliable diagnosis of DSM-5 premenstrual dysphoric disorder: The Carolina Premenstrual Assessment Scoring System (C-PASS). Am J Psychiatry.

[7] Eisenlohr-Moul TA, Schmalenberger KM, Owens SA, Peters JR, Dawson DN, Girdler SS (2018). Perimenstrual exacerbation of symptoms in borderline personality disorder: evidence from multilevel models and the Carolina Premenstrual Assessment Scoring System. Psychol Med.

[8] Peters W, Freeman MP, Kim S, Cohen LS, Joffe H (2017). Treatment of premenstrual breakthrough of depression with adjunctive oral contraceptive pills compared with placebo. J Clin Psychopharmacol.

[9] Halbreich U (2008). Selective serotonin reuptake inhibitors and initial oral contraceptives for the treatment of PMDD: effective but not enough. CNS Spectr.

[10] Nock MK, Kleiman EM, Abraham M, Bentley KH, Brent DA, Buonopane RJ, et al. Consensus statement on ethical & safety practices for conducting digital monitoring studies with people at risk of suicide and related behaviors. Psychiatr Res Clin Pract. 2020. 10.1176/appi.prcp.20200029. 10.1176/appi.prcp.20200029 PMC8372411 34414359

